# Predicting changes in glycemic control among adults with prediabetes from activity patterns collected by wearable devices

**DOI:** 10.1038/s41746-021-00541-1

**Published:** 2021-12-21

**Authors:** Mitesh S. Patel, Daniel Polsky, Dylan S. Small, Sae-Hwan Park, Chalanda N. Evans, Tory Harrington, Rachel Djaraher, Sujatha Changolkar, Christopher K. Snider, Kevin G. Volpp

**Affiliations:** 1 Ascension, 4600 Edmundson Rd, St. Louis, MO 63134 USA; 2 Perelman School of Medicine, University of Pennsylvania, 3400 Civic Center Blvd, Philadelphia, PA 19104 USA; 3 The Wharton School, University of Pennsylvania, 3730 Walnut St, Philadelphia, PA 19104 USA; 4 Johns Hopkins University, 624 N. Broadway, Baltimore, MD 21205 USA; 5 University of Michigan Medical School, 1301 Catherine St, Ann Arbor, MI 48109 USA; 6 Crescenz Veterans Affairs Medical Center, 3900 Woodland Ave, Philadelphia, PA 19104 USA

**Keywords:** Outcomes research, Diabetes

## Abstract

The use of wearables is increasing and data from these devices could improve the prediction of changes in glycemic control. We conducted a randomized trial with adults with prediabetes who were given either a waist-worn or wrist-worn wearable to track activity patterns. We collected baseline information on demographics, medical history, and laboratory testing. We tested three models that predicted changes in hemoglobin A1c that were continuous, improved glycemic control by 5% or worsened glycemic control by 5%. Consistently in all three models, prediction improved when (a) machine learning was used vs. traditional regression, with ensemble methods performing the best; (b) baseline information with wearable data was used vs. baseline information alone; and (c) wrist-worn wearables were used vs. waist-worn wearables. These findings indicate that models can accurately identify changes in glycemic control among prediabetic adults, and this could be used to better allocate resources and target interventions to prevent progression to diabetes.

Adults with diabetes have higher rates of cardiovascular disease, kidney failure, and death^1^. Nearly 90 million adults in the United States have prediabetes with an elevated blood glucose level that puts them at higher risk of developing diabetes in the future^2^. While behavioral interventions and medications have proven effective for preventing progression to diabetes, they are significantly underutilized^2^.

The effectiveness of diabetes prevention could be enhanced by more efficient targeting of resources for preventive interventions to adults that have the highest risk of developing diabetes in the near term. However, current risk prediction models vary in accuracy and most focus on predicting outcomes over longer-term periods such as 5–10 years^3–5^. Moreover, these models typically rely on information available at a single timepoint and do not account for levels or changes in daily health behaviors which are known to be associated with changes in glycemic control^6^.

Wearable devices are increasingly being adopted and could provide real-time access to data on physical activity, sleep, and heart rate patterns^7,8^. Risk prediction models that incorporate these data could improve near-term identification of adults with worsening glycemic control who are at the highest risk of developing diabetes. Wearables are most commonly worn on the wrist or waist, but differ in that waist-worn wearables are less expensive and collect less activity information. Body location may also change the accuracy of activity tracking and device utilization^8–12^. The ideal position for using a wearable device to inform risk prediction is unknown and needs further study.

In this study, our objective was to use a randomized trial to evaluate the use of data from waist-worn wearables vs. wrist-worn wearables to improve risk prediction models for changes in glycemic control among adults with prediabetes during a 6-month remote-monitoring period. We chose to conduct this comparison because these are the two most common sites for wearing an activity tracker and a randomized trial of real-world utilization of these devices provides a more pragmatic evaluation than a more controlled evaluation. We collected baseline information on demographics, medical history, and laboratory testing from all participants and then fit prediction models to evaluate the following comparisons: (a) machine learning methods vs. traditional regression models; (b) baseline information with wearable data vs. baseline information alone; (c) data from wrist-worn wearables vs. waist-worn wearables.

The trial protocol was approved by the University of Pennsylvania Institutional Review Board and the study was pre-registered on clinicaltrials.gov (NCT03544320). Potential participants were identified using the electronic health record at Penn Medicine and invited by email. Participants were eligible if they were age 18 years or older, provided informed consent, had a smartphone or tablet compatible with the wearables, and completed baseline laboratory testing with a hemoglobin A1c of 5.7 to 6.4. Participants completed surveys on their demographics, comorbidities, and other behaviors that have been shown to be associated with developing diabetes (smoking status, awareness of prediabetes, first degree relative with diabetes, and medications to control blood sugar). Participants were sent a digital weight scale to obtain a baseline weight using Skype or FaceTime with the study team to confirm their identity. Participants were given instructions on how to obtain baseline laboratory testing at no cost to them. All of this information collected at baseline was used in each of the models (detailed descriptions of each variable are available in the Methods section).

Participants were randomized electronically by stratifying on baseline HbA1c (5.7–6.0 or 6.1–6.4) and using block sizes of two to use a waist-worn wearable (Fitbit Zip) or wrist-worn wearable (Fitbit Charge 2 HR). The waist-worn wearable collected data on physical activity while the wrist-worn wearable collected data on physical activity, sleep, and heart rate. Participants authorized the Way to Health research technology platform at the University of Pennsylvania to collect data from Fitbit^13^. Participants were asked to use the wearable devices throughout the 6-month study and to sync their wearable with the Fitbit smartphone application daily. Participants who did not sync their devices for four consecutive days were sent an automated reminder to sync data with the smartphone application. At 6 months, all participants were asked to complete end-of-study laboratory testing and an end-of-study weigh-in.

The main outcome measure was the change in hemoglobin A1c. This was assessed in three ways. First, we evaluated continuous changes in hemoglobin A1c (primary outcome measure) using R squared. Second, we evaluated a worsening in glycemic control by creating a binary indicator to represent whether the hemoglobin A1c level increased by 0.3 points (about a 5% relative increase) using Area Under the ROC Curve (AUC). Third, we evaluated an improvement in glycemic control by creating a binary indicator to represent whether the hemoglobin A1c level decreased by 0.3 points (about a 5% relative reduction) using AUC. We selected 0.3 points as our cutoff because this 5% change has been used previously and prior research indicated that a change of this magnitude in either direction was associated with a significant change in the risk of progressing to diabetes^14–16^.

We fit six different modeling techniques. This included three regression-based models (ordinary regression without regularization, ridge regression, and lasso regression), two tree-based models (random forest and gradient boosting trees), and one ensemble model incorporating ridge regression, random forest, and gradient boosting. We present the findings from ordinary regression without regulation and ensemble machine learning. All other models are available in the Supplement and generally had similar prediction to traditional and machine learning techniques, respectively.

## Results

### Participant characteristics

The sample had a mean (SD) age of 56.7 years (12.7), body mass index of 32.7 (7.3) kg/m^2^, and baseline hemoglobin A1c of 6.1 (0.2); 69.4% were female, 18.8% were black, 2.7% were actively smoking, 50.5% had a first degree relative with diabetes, 91.4% were aware they had prediabetes, and 18.8% reported taking a medication for blood sugar control (Table 1). Characteristics were similar between the two arms (*P* values all > 0.05). Compared to participants that completed end-of-study laboratory testing, participants lost to follow-up were mostly similar but had higher baseline weight and lower baseline rate of hyperlipidemia (Supplementary Table 1).

**Table 1:** Characteristics of the participant sample.

	Waist-worn wearable (*N* = 93)	Wrist-worn wearable (*N* = 93)	Total (*n* = 186)
*Sociodemographics*			
Age, mean (SD), years	55.7 (13.1)	57.8 (12.1)	56.7 (12.7)
Female, N (%)	58 (62.4)	65 (69.9)	123 (66.1)
Race/ethnicity, N (%)			
White non-Hispanic	63 (67.7)	66 (71.0)	129 (69.4)
Black non-Hispanic	18 (19.4)	17 (18.3)	35 (18.8)
Asian non-Hispanic	4 (4.3)	4 (4.3)	8 (4.3)
Hispanic	5 (5.4)	4 (4.3)	9 (4.8)
Other	3 (3.2)	2 (2.2)	5 (2.7)
Education, N (%)			
High school graduate	22 (23.7)	26 (28.0)	48 (25.8)
Some college or specialized training	38 (40.9)	36 (38.7)	73 (39.2)
College graduate	33 (35.5)	31 (33.3)	64 (34.4)
Martial Status, N (%)			
Single	15 (16.1)	9 (9.7)	24 (12.9)
Married	67 (72.0)	65 (69.9)	132 (71.0)
Other	11 (11.8)	19 (20.4)	30 (16.1)
Annual household income, N (%)			
< $50,000	14 (15.1)	20 (21.5)	34 (18.3)
50,000 to 100,000	33 (35.5)	37 (39.8)	70 (37.6)
> 100,000	46 (49.5)	36 (38.7)	81 (43.5)
*Baseline measurements*			
Hemoglobin A1c, mean (SD)	6 (0.2)	6.1 (0.2)	6.1 (0.2)
Body mass index, mean (SD)	33.2 (7.0)	32.2 (7.7)	32.7 (7.3)
Weight, mean lbs. (SD)	208.3 (51.3)	199.8 (53.4)	204.1 (52.4)
LDL, mean (SD)	104.1 (31.6)	107.1 (33.7)	105.6 (32.6)
Smoking actively, N (%)	2 (2.2)	3 (3.2)	5 (2.7)
Hypertension, N (%)	39 (41.9)	43 (46.2)	82 (44.1)
Hyperlipidemia, N (%)	45 (48.4)	57 (61.3)	102 (54.8)
Charlson Comorbidity Index, median (IQR)	1 (0–2)	1 (0–2)	1 (0–2)
Taking medication for high blood sugar, N (%)	16 (17.2)	19 (20.4)	35 (18.8)
Taking medication for high cholesterol, N (%)	39 (41.9)	49 (52.7)	88 (47.3)
Aware of prediabetic status, N (%)	88 (94.6)	82 (88.2)	170 (91.4)
First degree relative diagnosed with diabetes, N (%)	47 (50.5)	47 (50.5)	94 (50.5)

### Hemoglobin A1c testing and activity measures

**Fig. 1:**
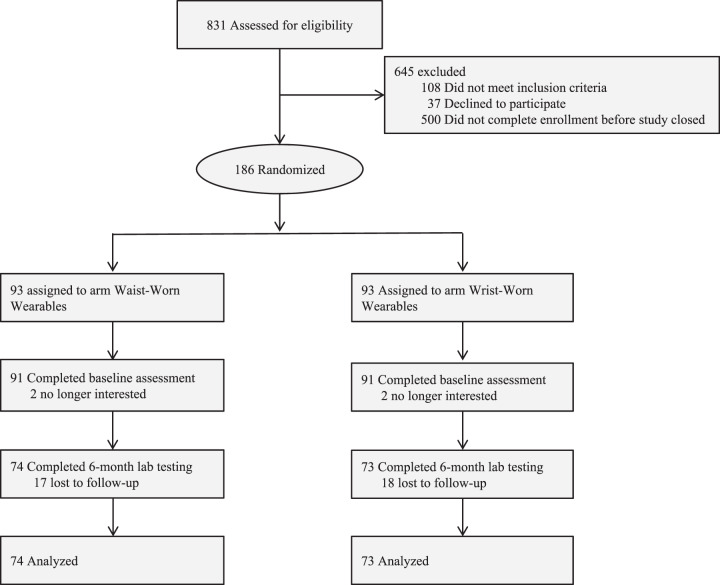
Study flow diagram. Displayed is the flow of patients for each arm of the trial.

In the waist-worn wearable arm, 74 of 93 (77.7%) participants obtained end-of-study laboratory testing (Fig. 1). Among these participants, the mean (SD) hemoglobin A1c was 6.0 (0.2) at baseline and 6.0 (0.3) at 6 months, 5 (6.8%) had increases in their hemoglobin A1c level of ≥0.3, and 14 (18.9%) had a decrease of ≥0.3 (Table 2). In the wrist-worn wearable arm, 73 of 93 (78.5%) participants obtained end-of-study laboratory testing (Fig. 1). Among these participants, the mean (SD) hemoglobin A1c was 6.1 (0.2) at baseline and 6.1 (0.3) at 6 months, 11 (15.1%) had increases in their hemoglobin A1c level of ≥0.3, and 12 (16.4%) had a decrease of ≥0.3 (Table 2). For all physical activity measures, mean and standard deviations of the data, along with rates of missing data, are available in Supplementary Table 2. For step counts, missing data rates were lower in the wrist-worn arm than the waist-worn arm (11.9% vs. 24.2%).

**Table 2:** Hemoglobin A1c and weight measures.

Outcome	Measure	Waist-Worn Wearable	Wrist-Worn Wearable
Hemoglobin A1c (HbA1c)	*Baseline*		
	Patients, N (%)	93 (100.0)	93 (100.0)
	HbA1c, mean (SD)	6.0 (0.2)	6.1 (0.2)
	HbA1c < 5.7, N (%)	0 (0.0)	0 (0.0)
	HbA1c 5.7 to 5.9, N (%)	35 (37.6)	29 (31.2)
	HbA1c 6.0 to 6.4, N (%)	58 (62.4)	64 (68.8)
	HbA1c > 6.4, N (%)	0 (0.0)	0 (0.0)
	*6 Months*		
	Patients, n (%)	74 (79.6)	73 (78.5)
	HbA1c, mean (SD)	6.0 (0.3)	6.1 (0.3)
	HbA1c < 5.7, N (%)	6 (8.1)	2 (2.7)
	HbA1c 5.7 to 5.9, N (%)	24 (32.4)	23 (31.5)
	HbA1c 6.0 to 6.4, N (%)	41 (55.4)	42 (57.5)
	HbA1c > 6.4, N (%)	3 (4.1)	6 (8.2)
	Proportion with HbA1c Increase of ≥0.3, N (%)	5 (6.8)	11 (15.1)
	Proportion with HbA1c Decrease of ≥0.3, N (%)	14 (18.9)	12 (16.4)
Weight, lbs.	*Baseline*		
	Patients, n (%)	93 (100.0)	93 (100.0)
	Lbs., mean (SD)	208.3 (51.3)	199.8 (53.4)
	*6 Months*		
	Patients, N (%)	76 (81.7)	82 (88.2)
	Lbs., mean (SD)	203.1 (46.5)	200.6 (56.1)
	Proportion with Weight Increase, N (%)	42 (55.3)	44 (53.7)
	Proportion with Weight Decrease, N (%)	33 (43.4)	35 (42.7)

### Continuous prediction model

**Fig. 2:**
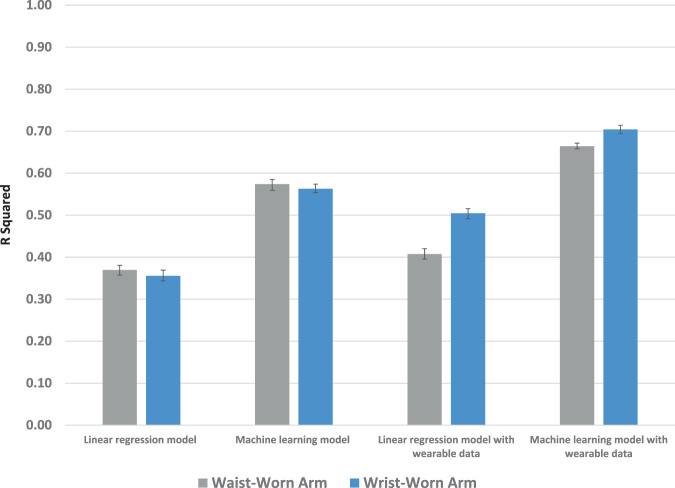
Prediction of continuous change in hemoglobin A1c. Displayed by type of model (linear regression or ensemble machine learning), use of data (with or without wearable data), and location of wearable (waist or wrist). Data presented are R squared and 95% confidence intervals from the testing set.

In the continuous model using the standard approach with only baseline information and traditional linear regression, there was no difference in prediction (R squared in waist-worn arm, 0.37, 95% CI = 0.357 to 0.380; R squared in wrist-worn arm, 0.36, 95% CI = 0.343 to 0.369; *P* for difference = 0.15). In the enhanced approach in which wearable data was added to traditional linear regression, the wrist-worn arm had significantly greater prediction (R squared in waist-worn arm, 0.41, 95% CI = 0.395 to 0.420; R squared in wrist-worn arm, 0.50, 95% CI = 0.491 to 0.515; *P* < 0.001). Prediction improved in this approach when ensemble machine learning was used with greater prediction in the wrist-worn arm (R squared in waist-worn arm, 0.66, 95% CI = 0.658 to 0.671; R squared in wrist-worn arm, 0.70, 95% CI = 0.694 to 0.714; *P* < 0.001) (Fig. 2).

### Binary prediction models

**Fig. 3:**
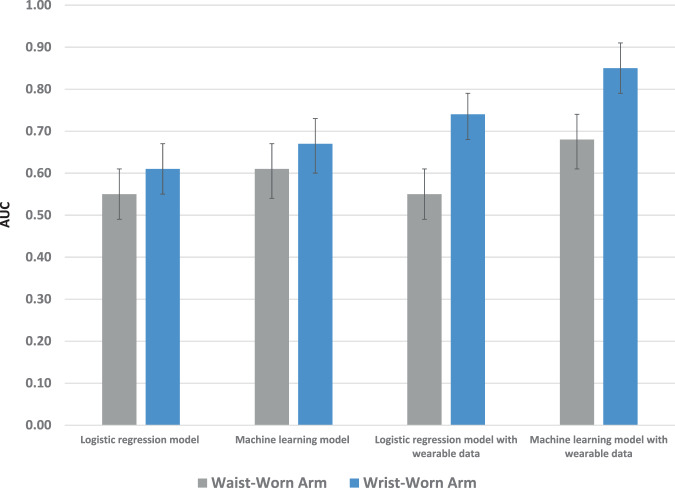
Prediction of hemoglobin A1c worsening. Represents prediction for an increase in hemoglobin A1c of ≥ 0.3. Displayed by type of model (logistic regression or ensemble machine learning), use of data (with or without wearable data), and type of wearable (waist or wrist). Data presented are Area Under the Curve (AUC) and 95% confidence intervals from the testing set.

In the binary model that evaluated a worsening of glycemic control, the standard approach with baseline information and traditional logit regression found no difference in prediction between arms (AUC in waist-worn arm, 0.55, 95% CI = 0.49–0.61; AUC in wrist-worn arm, 0.61, 95% CI = 0.55–0.67; *P* = 0.15). In the enhanced approach that added wearable data to the traditional logit regression, the wrist-worn arm had significantly greater prediction (AUC in waist-worn arm, 0.55, 95% CI = 0.48–0.61; AUC in wrist-worn arm, 0.74, 95% CI = 0.68–0.79; *P* < 0.001). Prediction improved in this approach when ensemble machine learning was used with greater prediction in the wrist-worn arm (AUC in waist-worn arm, 0.68, 95% CI = 0.61–0.74; AUC in wrist-worn arm, 0.85, 95% CI = 0.79–0.90; *P* < 0.001) (Fig. 3**)**.

**Fig. 4:**
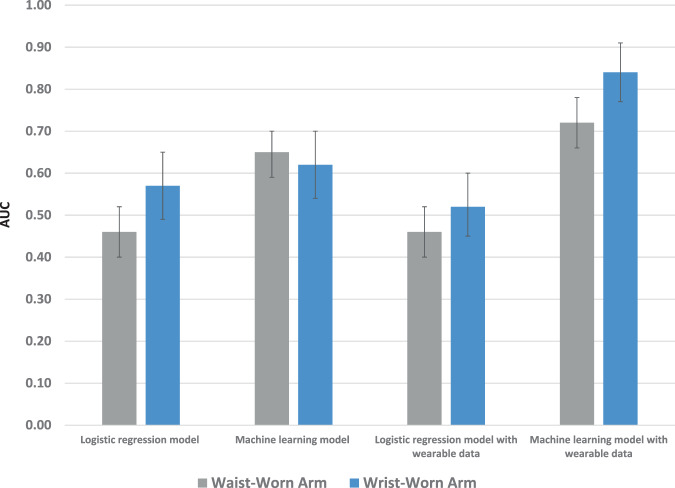
Prediction of hemoglobin A1c improvement. Represents prediction for a decrease in hemoglobin A1c of ≥ 0.3. Displayed by type of model (logistic regression or ensemble machine learning), use of data (with or without wearable data), and type of wearable (waist or wrist). Data presented are Area Under the Curve (AUC) and 95% confidence intervals from the testing set.

Results were similar in the binary model that evaluated improvement of glycemic control with the greatest prediction in the enhanced approach with wearable data that used ensemble prediction (AUC in waist-worn arm, 0.72, 95% CI = 0.66–0.77; AUC, 0.84, 95% CI = 0.77–0.91; *P* = 0.01) (Fig. 4**)**

Results from all modeling approaches are available in Supplementary Tables 3–17. Prediction in the enhanced models for the wrist-worn arm was similar when excluding sleep and heart rate data (Supplementary Tables 7, 12, 17) indicating that these variables may not be responsible for improved prediction in this arm. There were no reported adverse events in this trial.

## Discussion

In this randomized trial testing the use of data from different types of wearables to improve the prediction of changes in glycemic control, we found the best prediction when using ensemble machine learning methods with data from wrist-worn wearables. These findings were consistent whether we evaluated hemoglobin A1c changes continuously or based on an increase or decrease of ≥ 0.3 (glycemic control worsened or improved by 5%).

Improvements in prediction among the wrist-worn wearables arm were likely not due to the collection of sleep and heart rate patterns as our analyses found prediction was similar when these data were excluded. This indicates that superior prediction may have been due to either lower missing data rates in the wrist-worn arm or differences in accuracy between the two arms. On average, participants in the wrist-worn arm had about 1000 steps more than those in the waist-worn arm. This could have been because they were more active or they wore their devices for longer periods of the day and thereby had more accurate data collection. It is also important to note that there was more of an imbalance in the waist-worn arm in the proportion of participants with an increase vs. a decrease in a 5% change in hemoglobin A1c. Given the smaller sample size of this trial, this imbalance could have impacted prediction in this arm. Nonetheless, findings were consistent across all modeling approaches and sensitivity analyses.

Our study has limitations. First, it was conducted with a small sample of patients at one health system and therefore these approaches should be tested in other settings. Second, it was conducted over a 6-month period and longer studies are needed to determine if these shorter-term changes in hemoglobin A1c lead to longer-term changes in developing diabetes. Third, we only used hemoglobin A1c as the measure for glycemic control and did use other measures such as fasting glucose levels. Fourth, we did not capture information on diet which is an important factor in the progression from prediabetes to diabetes. We also did not utilize information on change in weight in the prediction models because this information would not traditionally be available from wearable devices alone. Future studies could consider collecting diet and weight information in prediction models.

Daily health behaviors contribute significantly toward longer-term health outcomes^6^, but most prior models predicting glycemic control among prediabetes have not accounted for these factors^3–5^. Wearable devices and smartphones can accurately track activity patterns and could help to address this limitation^7,8^. In this randomized trial, we found that consistently in all three models, prediction improved when (a) machine learning was used vs. traditional regression, with ensemble methods performing the best; (b) baseline information with wearable data was used vs. baseline information alone; and (c) wrist-worn wearables were used vs. waist-worn wearables.

## Methods

### Study design

PREDICT-DM (Prediction using a Randomized Evaluation of Data collection Integrated through Connected Technologies for Diabetes Mellitus) was a randomized clinical trial conducted between July 9, 2018 and August 1, 2019 consisting of 6-month remote-monitoring period. The trial protocol was approved by the University of Pennsylvania Institutional Review Board and the study was pre-registered on clinicaltrials.gov (NCT03544320).

The study was conducted using Way to Health^13^, a research technology platform at the University of Pennsylvania used previously for remote-monitoring of activity patterns^17–20^. Participants used the study website to create an account, provide informed consent online, and to complete baseline and validated survey assessments. Participants authorized Way to Health to collect activity data from the wearable devices for research purposes. Each participant chose whether to receive regular study communications by email, text message, or both. Participants were given instructions on how to obtain baseline laboratory testing for hemoglobin A1c and LDL-C (low-density lipoprotein cholesterol) at no cost to the participant. All participants received $25 for completing baseline laboratory testing, $50 for fully enrolling in the trial, and $100 for completing the 6-month study, laboratory testing at 6 months, and end-of-study surveys.

### Participants

The electronic health record at Penn Medicine was used to identify adults with a prior hemoglobin A1c of 5.7–6.4 who did not have a diagnosis of diabetes or a past hemoglobin A1c ≥ 6.5. The study team conducted outreach by email invitations to approximately 8000 patients and invited them to learn more about the study online.

Participants were eligible if they were age 18 years or older, able to read and provide informed consent to participate, owned a smartphone or tablet compatible with the wearable device, and had baseline hemoglobin A1c of 5.7–6.4 within the past 90 days. Participants were excluded if they reported that they had any medical conditions or reasons that would prohibit them from completing the 6-month study.

### Randomization

After completing baseline laboratory testing, participants were randomized electronically by stratifying on baseline HbA1c (5.7–6.0 or 6.1–6.4) and using block sizes of two. All investigators, statisticians, and data analysts were blinded to arm assignments until the study and analysis were completed. Participants were randomized to either the waist-worn (Fitbit Zip) or wrist-worn (Fitbit Charge HR 2) wearable arm. These devices were mailed to participants with instructions on how to set them up and provide access for the Way to Health research platform to obtain data on activity patterns from Fitbit. The study team was available by phone to help participants setup their devices.

### Interventions and follow-up

All participants received a digital weight scale (Etekcity Inc). A baseline weight was obtained by using Skype (Microsoft Inc.) or FaceTime (Apple Inc.) to identify the participant and document their weight while stepping on the scale.

Participants were asked to use the wearable devices throughout the 6-month study and to sync their wearable with the Fitbit smartphone application daily. Participants who did not sync their devices for four consecutive days were sent an automated reminder to sync data with the smartphone application.

At 6 months, all participants were asked to complete end-of-study laboratory testing at no cost to them including hemoglobin A1c and LDL-C and an end-of-study weigh-in using Skype or FaceTime to capture a weight from the digital scale.

### Statistical analysis

The main outcome measure was the change in hemoglobin A1c. This was assessed in three ways. First, we evaluated continuous changes in hemoglobin A1c (primary outcome measure). Second, we evaluated a worsening in glycemic control by creating a binary indicator to represent whether the hemoglobin A1c level increased by 0.3 points (about a 5% relative increase). Third, we evaluated an improvement in glycemic control by creating a binary indicator to represent whether the hemoglobin A1c level decreased by 0.3 points (about a 5% relative reduction).

We evaluated participants who had hemoglobin A1c levels collected at baseline and after the 6-month remote-monitoring intervention. Of the 186 randomized, 39 were lost to follow-up and did not have an end-of-study hemoglobin A1c. The final sample had 147 participants with complete data (74 of 93 in waist-worn wearables arm and 73 of 93 in wrist-worn wearables arm).

Based on previously published approaches^3–5^, we developed a standard model that comprised data available at baseline as follows. Demographic information was collected using surveys included age, gender, race/ethnicity, education, marital status, and annual household income. Surveys also asked participants if they actively smoked, had high blood pressure, had high cholesterol, had a first degree relative with diabetes, if they previously were aware they had prediabetes, and if they were actively taking a medication to control their blood sugar. We used the electronic health record to obtain the Charlson Comorbidity Index score which has been shown to predict 10-year mortality and can be used for risk adjustment^21^. Baseline hemoglobin A1c and weight were collected as previously described.

An enhanced model used the same data as in the standard model but also included information from the wearable devices as follows. Physical activity data included mean daily steps and mean minutes of moderate-to-vigorous physical activity (MVPA) for each of the 6 months of the study. Heart rate data included mean monthly levels of daily resting heart rate and daily minutes in which the heart rate was in the fatburn, cardio, or peak zone. Sleep data including mean monthly level of daily time asleep, number of times the participants awoke overnight, and sleep efficiency (calculated as the total time asleep divided by the total time in bed after initially falling asleep). To address missing wearable data, we used a two-fold approach. First, we included dichotomous indicator variables for each feature at participant-day level, which were aggregated by participant before feeding into machine learning training stage. Second, we conducted 10 imputations using the multiple imputations by chain equations (MICE) with a mixed effect model to impute missing data for better training effectiveness in tree-based algorithms preventing homogenous process in leaf-node developments for contextually heterogenous feature^22^. Predictors for imputation included demographics, comorbidities, baseline measures, and study month.

All the variables centered and standardized (i.e., 0 mean and 1 standard deviation) and then principal component analysis was used to reduce dimension size and engineer features for algorithm training^23,24^. After evaluation, we selected 16 principal components which captured 83% of covariance in the wrist-worn wearables arm and 91% of covariance in the waist-worn wearables arm.

The sample was split for each study arm into training and testing folds that were mutually exclusive at the participant level in a 3:2 ratio. To address shortcomings of low power due to small sample sizes in training sets, we applied kernel bootstrapping to each arm separately until 600 resamples (300 each arm) in the training set and 400 resample (200 each arm) in the test set were obtained. This approach has been used in a simulation study^25^ as well as empirical literature over various fields^26,27^.

We fit six different modeling techniques to the training set. This included three regression-based models (ordinary regression without regularization, ridge regression, and lasso regression), two tree-based models (random forest and gradient boosting trees), and one ensemble model incorporating ridge regression, random forest and gradient boosting. For the gradient boosting and random forest models, we used an exhaustive grid search (i.e., all grid points tested) and 5-fold cross validation on the training cohort to determine hyperparameters. For each of the different modeling combinations—each model (standard and enhanced), each trial arm, and each outcome (continuous HbA1c change and binary indicators of A1c increased or decreased)—hyperparameters separately tuned. The area under the Receiver Operating Characteristic curve and the R-squared score were used in valuations of hyperparameter tuning for regression and classification algorithms. Models were not recalibrated when applied to the testing set.

To assess model performance, they were applied to the holdout testing set. For the continuous model, we used the R squared score and estimated 95% confidence intervals using the corrected and accelerated bootstrapping method with 1,000 iterations, as first suggested by Efron^28^ and later validated by Carpenter and Bithell in the medical context^29^. Student’s *t* test was used to assess for differences between comparison groups using the 1,000 bootstraps. For the binary outcome models, we used the area under the receiver operating characteristic curve (AUC). We used a nonparametric comparison test for differences between comparison groups^30^. In an exploratory analysis, we conducted prediction for the wrist-worn arm but without including sleep and heart rate data which are not collected by the waist-worn arm. This helps to understand whether it was these data elements or other factors that explain differences in prediction based on location site.

### Reporting summary

Further information on research design is available in the Nature Research Reporting Summary linked to this article.

## Supplementary information

Supplementary Information

Reporting Summary

## Data Availability

Data are not available because they contain sensitive patient information from electronic health records. De-identified data and statistical code are available upon request to the corresponding author.
