# Supplementary material for: Predicting changes in glycemic control among adults with prediabetes from activity patterns collected by wearable devices
Source: NPJ Digit Med. 2021 Dec 21;4:172. doi: 10.1038/s41746-021-00541-1 (PMC8692591; doi:10.1038/s41746-021-00541-1)
Supplement: Supplementary file 1 — Supplementary Information [file 41746_2021_541_MOESM1_ESM.pdf]

Supplementary Information for

Predicting Changes in Glycemic Control among Adults with Prediabetes from Activity Patterns Collected by Wearable Devices

Table of Contents

Supplementary Table 1. Characteristics of the Participant Sample by Follow-Up Status

Supplementary Table 2. Wearable Measures and Missing Data.

Supplementary Table 3. Standard models without wearable data for a hemoglobin A1c continuous change.

Supplementary Table 4. Enhanced models with wearable data for a hemoglobin A1c continuous change.

Supplementary Table 5. Standard and enhanced models for the waist-worn arm for a hemoglobin A1c continuous change.

Supplementary Table 6. Standard and enhanced models for the wrist-worn arm for a hemoglobin A1c continuous change.

Supplementary Table 7. Standard and enhanced models for the wrist-worn arm for a hemoglobin A1c continuous change but excluding sleep and heart rate data.

Supplementary Table 8. Standard models without wearable data for a hemoglobin A1c increase of 0.3 or greater.

Supplementary Table 9. Enhanced models with wearable data for a hemoglobin A1c increase of 0.3 or greater.

Supplementary Table 10. Standard and enhanced models for the waist-worn arm for a hemoglobin A1c increase of 0.3 or greater.

Supplementary Table 11. Standard and enhanced models for the wrist-worn arm for a hemoglobin A1c increase of 0.3 or greater.

Supplementary Table 12. Standard and enhanced models for the wrist-worn arm for a hemoglobin A1c increase of 0.3 or greater but excluding sleep and heart rate data.

Supplementary Table 13. Standard models without wearable data for a hemoglobin A1c decrease of 0.3 or greater.

Supplementary Table 14. Enhanced models with wearable data for a hemoglobin A1c decrease of 0.3 or greater.

Supplementary Table 15. Standard and enhanced models for the waist-worn arm for a hemoglobin A1c decrease of 0.3 or greater.
Supplementary Table 16. Standard and enhanced models for the wrist-worn arm for a hemoglobin A1c decrease of 0.3 or greater.

Supplementary Table 17. Standard and enhanced models for the wrist-worn arm for a hemoglobin A1c decrease of 0.3 or greater but excluding sleep and heart rate data.

Supplementary Table 1. Characteristics of the Participant Sample by Follow-Up Status

	Completed 6-Month HbA1c (N=147)	Lost to Follow-Up (N=39)	*P* value
**Sociodemographics**			
Age, mean (SD), years	57.5 (12.2)	53.8 (14.0)	0.07
Female, N (%)	47 (32.0)	16 (41.0)	0.29
Race/ethnicity, N (%)			0.31
White non-Hispanic	99 (67.3)	30 (76.9)	
Black non-Hispanic	30 (20.4)	5 (12.8)	
Asian non-Hispanic	8 (5.4)	0 (0.0)	
Hispanic	6 (4.1)	3 (7.7)	
Other	4 (2.7)	1 (2.6)	
Education, N (%)			0.12
High school graduate	41 (27.9)	7 (17.9)	
Some college or specialized training	59 (40.1)	15 (38.5)	
College graduate	47 (32.0)	17 (43.6)	
Martial Status, N (%)			0.79
Single	18 (12.2)	6 (15.4)	
Married	107 (72.8)	25 (64.1)	
Other	22 (15.0)	8 (20.5)	
Annual household income, N (%)			0.76
< $50,000	27 (18.4)	7 (17.9)	
50,000 to 100,000	54 (36.7)	16 (41.0)	
> 100,000	66 (44.9)	16 (41.0)	
**Baseline Measurements**			
Hemoglobin A1c, mean (SD)	6.1 (0.2)	6.0 (0.2)	0.69
Body mass index, mean (SD)	32.1 (6.7)	34.9 (9.2)	0.08
Weight, mean lbs. (SD)	199.3 (47.8)	222.0 (64.4)	0.04
LDL, mean (SD)	105.6 (30.9)	107.0 (40.1)	0.99
Smoking actively, No. (%)	3 (2.0)	2 (5.1)	0.29
Hypertension, No. (%)	63 (43.2)	19 (50.0)	0.60
Hyperlipidemia, No. (%)	85 (58.6)	17 (50.0)	0.04
Charlson Comorbidity Index, median (IQR)	1 (0-2)	1 (0-2)	0.27
Taking medication for high blood sugar	29 (19.7)	6 (15.4)	0.54
Taking medication for high cholesterol	70 (47.6)	18 (46.2)	0.87
Aware of pre-diabetic status	133 (90.5)	37 (94.9)	0.39
First degree relative diagnosed with diabetes	70 (47.6)	24 (61.5)	0.12

Supplementary Table 2. Wearable Measures and Missing Data.

	Waist-Worn Wearable	Wrist-Worn Wearable
**Steps**		
Observations	16740	16740
Missing data, n (%)	4048 (24.2)	1984 (11.9)
Mean (SD)	6274.7 (4544.4)	7283.6 (4166.0)
**Minutes of MVPA**		
Observations	16740	16740
Missing data, n (%)	7083 (42.3)	5062 (30.2)
Mean (SD)	19.0 (26.5)	17.2 (22.4)
**Resting Heart Rate**		
Observations	NA	16740
Missing data, n (%)	NA	1821 (10.9)
Mean (SD)	NA	67.7 (9.5)
**Minutes Heart Rate in Fatburn**		
Observations	NA	16740
Missing data, n (%)	NA	1476 (8.8)
Mean (SD)	NA	447.3 (314.1)
**Minutes Heart Rate in Cardio**		
Observations	NA	16740
Missing data, n (%)	NA	1476 (8.8)
Mean (SD)	NA	41.3 (75.9)
**Minutes Heart Rate in Peak Zone**		
Observations	NA	16740
Missing data, n (%)	NA	1476 (8.8)
Mean (SD)	NA	2.7 (8.2)
**Minutes of Sleep**		
Observations	NA	16740
Missing data, n (%)	NA	3265 (19.5)
Mean (SD)	NA	435.8 (127.0)
**Times Awoke Per Night**		
Observations	NA	16740
Missing data, n (%)	NA	3265 (19.5)
Mean (SD)	NA	2.2 (1.9)
**Sleep Efficiency**		
Observations	NA	16740
Missing data, n (%)	NA	3265 (19.5)
Mean (SD)	NA	93.3 (4.9)

Supplementary Table 3. Standard models without wearable data for a hemoglobin A1c continuous change.

Standard models without wearable data (Hemoglobin A1c Continuous Change)	Waist-Worn Arm R Squared (95% CI)	Wrist-Worn Arm R Squared (95% CI)	P-value
Linear regression	0.37 (0.357,0.380)	0.36 (0.343,0.369)	0.15
Lasso regression	0.49 (0.482,0.499)	0.50 (0.493,0.511)	0.06
Ridge regression	0.49 (0.483,0.500)	0.50 (0.494,0.512)	0.05
Classification and regression trees (CART)	0.07 (0.037,0.108)	0.10 (0.078,0.133)	0.12
Random forest	0.56 (0.547,0.564)	0.54 (0.530,0.551)	0.04
Gradient boosting	0.54 (0.518,0.554)	0.55 (0.529,0.562)	0.38
Ensemble machine learning	0.57 (0.559,0.585)	0.56 (0.554,0.574)	0.22

Supplementary Table 4. Enhanced models with wearable data for a hemoglobin A1c continuous change.

Enhanced models with wearable data (Hemoglobin A1c Continuous Change)	Waist-Worn Arm R Squared (95% CI)	Wrist-Worn Arm R Squared (95% CI)	P-value
Linear regression	0.41 (0.395,0.420)	0.50 (0.491,0.515)	<0.001
Lasso regression	0.62 (0.610,0.627)	0.68 (0.669,0.683)	<0.001
Ridge regression	0.62 (0.610,0.626)	0.68 (0.669,0.684)	<0.001
Classification and regression trees (CART)	0.26 (0.237,0.276)	0.35 (0.321,0.382)	<0.001
Random forest	0.65 (0.640,0.653)	0.69 (0.677,0.694)	<0.001
Gradient boosting	0.65 (0.644,0.660)	0.68 (0.665,0.686)	<0.001
Ensemble machine learning	0.66 (0.658,0.671)	0.70 (0.694,0.714)	<0.001

Supplementary Table 5. Standard and enhanced models for the waist-worn arm for a hemoglobin A1c continuous change.

Waist-Worn Arm (Hemoglobin A1c Continuous Change)	Standard Model R Squared (95% CI)	Enhanced Model R Squared (95% CI)	P-value
Linear regression	0.37 (0.357,0.380)	0.41 (0.395,0.420)	<0.001
Lasso regression	0.49 (0.482,0.499)	0.62 (0.610,0.627)	<0.001
Ridge regression	0.49 (0.483,0.500)	0.62 (0.610,0.626)	<0.001
Classification and regression trees (CART)	0.07 (0.037,0.108)	0.26 (0.237,0.276)	<0.001
Random forest	0.56 (0.547,0.564)	0.65 (0.640,0.653)	<0.001
Gradient boosting	0.54 (0.518,0.554)	0.65 (0.644,0.660)	<0.001
Ensemble machine learning	0.57 (0.559,0.585)	0.66 (0.658,0.671)	<0.001

Supplementary Table 6. Standard and enhanced models for the wrist-worn arm for a hemoglobin A1c continuous change.

Wrist-Worn Arm (Hemoglobin A1c Continuous Change)	Standard Model R Squared (95% CI)	Enhanced Model R Squared (95% CI)	P-value
Linear regression	0.36 (0.343,0.369)	0.50 (0.491,0.515)	<0.001
Lasso regression	0.50 (0.493,0.511)	0.68 (0.669,0.683)	<0.001
Ridge regression	0.50 (0.494,0.512)	0.68 (0.669,0.684)	<0.001
Classification and regression trees (CART)	0.10 (0.078,0.133)	0.35 (0.321,0.382)	<0.001
Random forest	0.54 (0.530,0.551)	0.69 (0.677,0.694)	<0.001
Gradient boosting	0.55 (0.529,0.562)	0.68 (0.665,0.686)	<0.001
Ensemble machine learning	0.56 (0.554,0.574)	0.70 (0.694,0.714)	<0.001

Supplementary Table 7. Standard and enhanced models for the wrist-worn arm for a hemoglobin A1c continuous change but excluding sleep and heart rate data.

Wrist-Worn Arm w/o Sleep & HR (Hemoglobin A1c Continuous Change)	Standard Model R Squared (95% CI)	Enhanced Model R Squared (95% CI)	P-value
Linear regression	0.36 (0.343,0.369)	0.39 (0.376,0.402)	0.001
Lasso regression	0.50 (0.493,0.511)	0.60 (0.597,0.612)	<0.001
Ridge regression	0.50 (0.494,0.512)	0.60 (0.596,0.611)	<0.001
Classification and regression trees (CART)	0.07 (0.046,0.106)	0.39 (0.355,0.411)	<0.001
Random forest	0.54 (0.527,0.548)	0.67 (0.664,0.682)	<0.001
Gradient boosting	0.54 (0.527,0.561)	0.67 (0.654,0.682)	<0.001
Ensemble machine learning	0.56 (0.551,0.571)	0.70 (0.686,0.709)	<0.001

Supplementary Table 8. Standard models without wearable data for a hemoglobin A1c increase of 0.3 or greater.

Standard models without wearable data (Hemoglobin A1c Increase ≥ 0.3)	Waist-Worn Arm AUC (95% CI)	Wrist-Worn Arm AUC (95% CI)	P-value
Logit	0.55 (0.49,0.61)	0.61 (0.55,0.67)	0.15
Lasso logit	0.61 (0.54,0.67)	0.67 (0.60,0.73)	0.21
Ridge logit	0.61 (0.55,0.67)	0.66 (0.60,0.73)	0.29
Classification and Regression Trees (CART)	0.50 (0.46,0.54)	0.61 (0.55,0.66)	<0.001
Random forest	0.58 (0.52,0.65)	0.66 (0.59,0.72)	0.05
Gradient boosting	0.59 (0.53,0.65)	0.62 (0.55,0.68)	0.77
Ensemble machine learning	0.61 (0.54,0.67)	0.67 (0.60,0.73)	0.21

Supplementary Table 9. Enhanced models with wearable data for a hemoglobin A1c increase of 0.3 or greater.

Enhanced models with wearable data (Hemoglobin A1c Increase ≥ 0.3)	Waist-Worn Arm AUC (95% CI)	Wrist-Worn Arm AUC (95% CI)	P-value
Logit	0.55 (0.48,0.61)	0.74 (0.68,0.79)	<0.001
Lasso logit	0.68 (0.62,0.74)	0.84 (0.79,0.89)	<0.001
Ridge logit	0.68 (0.61,0.74)	0.84 (0.79,0.89)	<0.001
Classification and Regression Trees (CART)	0.56 (0.52,0.61)	0.69 (0.64,0.74)	<0.001
Random forest	0.64 (0.58,0.71)	0.84 (0.78,0.90)	<0.001
Gradient boosting	0.70 (0.63,0.75)	0.85 (0.80,0.90)	<0.001
Ensemble machine learning	0.68 (0.61,0.74)	0.85 (0.79,0.90)	<0.001

Supplementary Table 10. Standard and enhanced models for the waist-worn arm for a hemoglobin A1c increase of 0.3 or greater.

Waist-Worn Arm (Hemoglobin A1c Increase ≥ 0.3)	Standard Model AUC (95% CI)	Enhanced Model AUC (95% CI)	P-value
Logit	0.55 (0.49,0.61)	0.55 (0.49,0.61)	0.97
Lasso logit	0.61 (0.54,0.67)	0.68 (0.61,0.74)	0.01
Ridge logit	0.61 (0.55,0.67)	0.68 (0.62,0.74)	0.01
Classification and Regression Trees (CART)	0.50 (0.46,0.54)	0.56 (0.52,0.61)	0.04
Random forest	0.58 (0.52,0.65)	0.64 (0.57,0.71)	0.09
Gradient boosting	0.59 (0.53,0.65)	0.70 (0.63,0.75)	0.003
Ensemble machine learning	0.61 (0.54,0.67)	0.68 (0.61,0.74)	0.02

Supplementary Table 11. Standard and enhanced models for the wrist-worn arm for a hemoglobin A1c increase of 0.3 or greater.

Wrist-Worn Arm (Hemoglobin A1c Increase ≥ 0.3)	Standard Model AUC (95% CI)	Enhanced Model AUC (95% CI)	P-value
Logit	0.61 (0.55,0.67)	0.74 (0.68,0.79)	0.0012
Lasso logit	0.67 (0.60,0.73)	0.84 (0.79,0.89)	<0.001
Ridge logit	0.66 (0.60,0.73)	0.84 (0.78,0.89)	<0.001
Classification and Regression Trees (CART)	0.61 (0.55,0.66)	0.69 (0.64,0.75)	0.0248
Random forest	0.66 (0.59,0.72)	0.84 (0.78,0.90)	<0.001
Gradient boosting	0.62 (0.55,0.68)	0.85 (0.80,0.90)	<0.001
Ensemble machine learning	0.67 (0.60,0.73)	0.85 (0.79,0.91)	<0.001

Supplementary Table 12. Standard and enhanced models for the wrist-worn arm for a hemoglobin A1c increase of 0.3 or greater but excluding sleep and heart rate data.

Wrist-Worn Arm w/o Sleep & HR (Hemoglobin A1c Increase ≥ 0.3)	Standard Model AUC (95% CI)	Enhanced Model AUC (95% CI)	P-value
Logit	0.61 (0.55,0.67)	0.72 (0.65,0.77)	0.010
Lasso logit	0.67 (0.60,0.73)	0.78 (0.71,0.83)	0.015
Ridge logit	0.66 (0.60,0.73)	0.78 (0.72,0.84)	0.009
Classification and Regression Trees (CART)	0.61 (0.55,0.66)	0.63 (0.58,0.68)	0.597
Random forest	0.66 (0.59,0.72)	0.83 (0.76,0.88)	<0.001
Gradient boosting	0.62 (0.55,0.68)	0.82 (0.75,0.87)	<0.001
Ensemble machine learning	0.67 (0.60,0.73)	0.84 (0.77,0.89)	<0.001

Supplementary Table 13. Standard models without wearable data for a hemoglobin A1c decrease of 0.3 or greater.

Standard models without wearable data (Hemoglobin A1c Decrease ≥ 0.3)	Waist-Worn Arm AUC (95% CI)	Wrist-Worn Arm AUC (95% CI)	P-value
Logit	0.46 (0.40,0.52)	0.57 (0.49,0.65)	0.01
Lasso logit	0.63 (0.57,0.69)	0.61 (0.53,0.69)	0.50
Ridge logit	0.63 (0.57,0.69)	0.60 (0.52,0.67)	0.21
Classification and Regression Trees (CART)	0.56 (0.51,0.60)	0.48 (0.43,0.53)	0.02
Random forest	0.67 (0.61,0.72)	0.61 (0.52,0.69)	0.04
Gradient boosting	0.63 (0.58,0.69)	0.62 (0.54,0.69)	0.51
Ensemble machine learning	0.65 (0.59,0.70)	0.62 (0.54,0.70)	0.53

Supplementary Table 14. Enhanced models with wearable data for a hemoglobin A1c decrease of 0.3 or greater.

Enhanced models with wearable data (Hemoglobin A1c Decrease ≥ 0.3)	Waist-Worn Arm AUC (95% CI)	Wrist-Worn Arm AUC (95% CI)	P-value
Logit	0.46 (0.41,0.52)	0.53 (0.45,0.60)	0.17
Lasso logit	0.69 (0.64,0.74)	0.80 (0.73,0.87)	0.02
Ridge logit	0.69 (0.63,0.74)	0.80 (0.72,0.87)	0.02
Classification and Regression Trees (CART)	0.55 (0.50,0.60)	0.59 (0.53,0.66)	0.29
Random forest	0.71 (0.65,0.76)	0.84 (0.77,0.90)	0.003
Gradient boosting	0.69 (0.63,0.74)	0.81 (0.74,0.87)	0.01
Ensemble machine learning	0.72 (0.66,0.77)	0.84 (0.77,0.91)	0.01

Supplementary Table 15. Standard and enhanced models for the waist-worn arm for a hemoglobin A1c decrease of 0.3 or greater.

**Waist-Worn Arm (Hemoglobin A1c Decrease ≥ 0.3)**	**Standard Model AUC (95% CI)**	**Enhanced Model AUC (95% CI)**	**P-value**
Logit	0.46 (0.40,0.52)	0.46 (0.40,0.52)	0.998
Lasso logit	0.63 (0.57,0.69)	0.69 (0.63,0.74)	0.02
Ridge logit	0.63 (0.57,0.69)	0.69 (0.64,0.74)	0.02
Classification and Regression Trees (CART)	0.56 (0.51,0.60)	0.55 (0.50,0.60)	0.80
Random forest	0.67 (0.61,0.72)	0.71 (0.65,0.76)	0.09
Gradient boosting	0.63 (0.58,0.69)	0.69 (0.63,0.74)	0.06
Ensemble machine learning	0.65 (0.59,0.70)	0.72 (0.66,0.78)	0.003

Supplementary Table 16. Standard and enhanced models for the wrist-worn arm for a hemoglobin A1c decrease of 0.3 or greater.

**Wrist-Worn Arm (Hemoglobin A1c Decrease ≥ 0.3)**	**Standard Model AUC (95% CI)**	**Enhanced Model AUC (95% CI)**	**P-value**
Logit	0.57 (0.49,0.65)	0.52 (0.45,0.60)	0.38
Lasso logit	0.61 (0.53,0.69)	0.80 (0.72,0.87)	<0.001
Ridge logit	0.60 (0.52,0.67)	0.80 (0.73,0.87)	<0.001
Classification and Regression Trees (CART)	0.48 (0.43,0.53)	0.59 (0.53,0.66)	0.003
Random forest	0.61 (0.52,0.69)	0.84 (0.76,0.90)	<0.001
Gradient boosting	0.62 (0.54,0.69)	0.81 (0.74,0.87)	<0.001
Ensemble machine learning	0.62 (0.54,0.70)	0.84 (0.77,0.91)	<0.001

Supplementary Table 17. Standard and enhanced models for the wrist-worn arm for a hemoglobin A1c decrease of 0.3 or greater but excluding sleep and heart rate data.

**Wrist-Worn Arm w/o Sleep & HR (Hemoglobin A1c Decrease ≥ 0.3)**	**Standard Model AUC (95% CI)**	**Enhanced Model AUC (95% CI)**	**P-value**
Logit	0.57 (0.49,0.65)	0.51 (0.43,0.60)	0.3235
Lasso logit	0.61 (0.53,0.69)	0.80 (0.72,0.87)	<0.001
Ridge logit	0.60 (0.52,0.67)	0.80 (0.72,0.87)	<0.001
Classification and Regression Trees (CART)	0.48 (0.43,0.53)	0.64 (0.57,0.71)	<0.001
Random forest	0.61 (0.52,0.69)	0.87 (0.80,0.92)	<0.001
Gradient boosting	0.62 (0.54,0.69)	0.85 (0.76,0.91)	<0.001
Ensemble machine learning	0.62 (0.54,0.70)	0.87 (0.78,0.93)	<0.001
